# A scoping review of oral language and social communication abilities in children with Tourette syndrome

**DOI:** 10.1111/1460-6984.12949

**Published:** 2023-09-04

**Authors:** Angela Feehan, Monique Charest

**Affiliations:** ^1^ Faculty of Rehabilitation Medicine University of Alberta Edmonton Alberta Canada

**Keywords:** language development, language disorders, scoping review, social communication, Tourette syndrome

## Abstract

**Background:**

Children with Tourette syndrome (TS) have historically experienced problems in academic and social settings, yet their language and communication abilities have not been extensively researched.

**Aims:**

This scoping review maps the literature on the oral language and social communication abilities of children with TS in order to describe the nature of the current literature, present a summary of major findings and identify where gaps exist.

**Methods:**

A scoping review was completed to identify studies measuring the oral language or social communication abilities of children with TS. A systematic search of six electronic databases was conducted to obtain published and unpublished literature. All English studies measuring the oral language or social communication abilities of children with TS were included. Information was extracted from records and knowledge was synthesised in a narrative summary.

**Main Contribution:**

We identified 56 records for inclusion. Almost all records were located in journals within the fields of psychology and psychiatry. Skills most often studied were verbal IQ and verbal fluency. The literature suggests an increased prevalence of language disorders and social communication problems in children with TS; however, literature comprehensively detailing these challenges was scarce. Language strengths were identified in verbal intelligence, story/sentence recall, categorisation and performance on tasks at the single‐word level.

**Conclusions:**

Oral language and social communication skills are important for academic and social success. This review brings scattered literature together to provide up‐to‐date information about language in children with TS and highlights that there are considerable gaps in our knowledge about language and communication in this population. This scoping review can inform future research and support speech language pathologists in the assessment of young people with TS.

**WHAT THIS PAPER ADDS:**

**What is already known on the subject**

- Speech‐language pathologists (SLPs) working in various contexts (e.g., schools, mental health teams) are likely to encounter children with Tourette syndrome (TS); however, the description of this population and potential communication characteristics is not well represented in the SLP literature. Previous literature reviews have reported strengths in verbal fluency and morphological processing. Challenges in expressive language, higher order language, social cognition and a propensity towards autistic traits have also been identified.

**What this paper adds to existing knowledge**

- This review differs from previous narrative reviews by employing a systematic approach to searching for literature. As a result, we identified 25 additional studies that had not been cited in previous reviews and additional relevant findings in 23 previously reviewed studies. This review confirms several previous conclusions about language in children with TS and extends or clarifies several others, thereby providing the most current information on oral language and social communication abilities. The use of current taxonomies of language and social communication helps to organise this literature for clinicians and researchers in speech‐language pathology and identifies a need for further research from the SLP perspective.

**What are the potential or actual clinical implications of this work?**

- These results imply that SLPs should screen children with TS for language disorders and investigate social communication and social interaction development. Clinicians can expect greater challenges in language and communication development for children with complex forms of TS (i.e., those who exhibit co‐occurring conditions such as attention‐deficit/hyperactivity disorder). The multidisciplinary nature of the current literature implies that clinical collaboration with other disciplines will be of particular benefit to serving this group of children.

## INTRODUCTION

Tourette syndrome (TS) is a neurodevelopmental disorder involving one or more vocal tics and multiple motor tics (American Psychiatric Association, APA, 2013). Tics can be simple, such as grunting (vocal) or eye blinking (motor), or complex, such as saying words (vocal) or completing a series of gestures (motor). TS is associated with several neurobiological and neurochemical differences, including reduced caudate volumes, cortical thickening, increased volumes of the corpus callosum (Greene et al., 2013) and dysfunction of the dopaminergic system (Singer, 2013). The prevalence of TS in children is estimated to be between 0.2% and 0.3% (Bisko et al., 2022), and boys are affected more than girls by a factor of 3.5 (Baizabal‐Carvallo & Jankovic, 2022). Tics begin in childhood, peak in severity between 10 and 12 years of age and wane in adulthood (Bloch, 2013). In about 80% of cases, TS co‐occurs with one or more co‐occurring conditions, such as attention deficit/hyperactivity disorder (ADHD), obsessive compulsive disorder (OCD) and anxiety and depression (Martino & Leckman, 2013). Quality of life has sometimes been reported as low for individuals with TS (e.g., Marek, 2006).

### Motivation and background to the review

Existing literature reports difficulties in areas of academic, neuropsychological and social function in children with TS and subtle challenges in aspects of social cognition in adults with TS. These areas of noted difficulty are all closely connected to language and communication function and therefore invite a closer look at language and communication skills directly. A summary of findings in these areas of development provides context for the review.

#### Summary of academic, neuropsychological and social development in children with TS

Children with TS have been found to have high rates of learning disabilities and these challenges sometimes are in areas of language development (i.e., writing, spelling and reading; Claussen et al., 2018; Mahone et al., 2002). Despite this, the relationship between language learning and learning disabilities in children with TS has received surprisingly little research attention.

Investigations into the neuropsychological characteristics of children with TS have explored aspects of cognition that are important for organising and regulating communication interactions, specifically, attention, memory and executive functioning (Hyter, 2017). Findings in children with TS point to challenges in divided and sustained attention (Chang et al., 2007; Sherman et al., 1998), long‐term non‐verbal memory (e.g., Bloch et al, 2006), inhibitory control (Morand‐Beaulieu et al., 2017a) and executive control (Sikora et al., 2019).

Several studies have found that children with TS experience lower social functioning compared to peers without TS (Stokes et al., 1991; Sukhodolsky et al., 2003; Zhu et al., 2006). When rated by their peers, children with TS may be perceived as more withdrawn, more aggressive and less likable (Bawden et al., 1998; Stokes et al., 1991). Some studies have reported difficulties in making and keeping friends and developing secure attachments (Champion et al., 1988; O'Hare et al., 2015). Many factors are likely to influence the social experience of children with TS; however, the presence of problems in social function indicates that we need to know more about how children with TS manage communication in social interactions. Social communication skills are necessary in social interactions and therefore tie in closely with social functioning.

#### Social cognition in adults with TS

Individuals draw on social cognitive skills during social communication and these skills are often considered foundational to social communication (e.g., Adams., 2005; Hyter, 2017). The research programme led by Eddy and colleagues in the early 2010s (e.g., Eddy et al., 2010a, 2010b, 2011) has documented that adults with TS process social information differently (see Eddy et al., 2017). Furthermore, socially inappropriate behaviour is a well‐known aspect of TS (i.e., cursing tics and non‐obscene socially inappropriate symptoms; NOSIS), which, along with social cognitive challenges, may indicate that pragmatic language challenges are a core feature of TS (Albin, 2018; Eddy, 2021; Eddy & Cavanna, 2013; Eddy et al., 2011). Studies of adults with TS have reported subtle challenges in several aspects of social cognition that tie into social communication, including understanding the appropriate use of nonliteral language (i.e., sarcasm and metaphor), reading intentions, understanding humour, understanding emotional states, understanding and considering others’ perspectives or mental states, social problem solving, social reasoning and recognising social indiscretions (Channon et al., 2003a; Channon et al., 2012; Eddy & Cavana, 2015; Eddy et al., 2010a, 2010b, 2011, 2015). Across these studies, co‐occurring diagnoses were present in a minority of participants, indicating that social cognitive differences may be central to TS. There are also some studies reporting no differences compared to healthy controls on social cognitive tasks involving theory of mind, empathy, identification of mental states and comprehension of emotional cues (Baron‐Cohen et al., 1997; Channon et al., 2004; Devinsky et al., 1993). The nuances of specific task demands and task difficulty may help to explain these inconsistent findings. Nonetheless, the accumulated findings suggest that social cognitive skills need to be explored more thoroughly in children with TS as they are relevant to social communication development in childhood.

#### Previous reviews of language and social communication in TS

These observations of challenges in aspects of social and cognitive functioning suggest that speech‐language pathologists (SLPs) may have a role in supporting language and communication in individuals with TS. This has motivated us to gather and organise literature that considers the full scope of oral language and social communication from a speech‐language pathology perspective. Knowledge on this topic can serve to highlight the potential role of SLPs in supporting children with TS and can help direct future research.

Several previous reviews have addressed aspects of language and communication in TS (Burd et al., 2008; De Nil et al., 2006; Morand‐Beaulieu et al., 2017b, Murphy & Eddy, 2013), with the most recent being a book chapter focused on social communication (Eddy, 2021). Eddy (2021) summarised several studies of language and social cognitive abilities, and outlined how tics and other behaviours in TS can violate conversational rules and interfere with communication. Eddy highlighted the need for a greater understanding of social challenges in TS from a pragmatic point of view, discussing several theories on the relationship between TS and social communication.

Earlier reviews have suggested that expressive language may be an area of challenge for individuals with TS (Burd et al., 2008; De Nil et al., 2006; Murphy & Eddy, 2013); however, specific strengths in expressive language have also been reported: verbal fluency, stem completion, naming and past tense production speed (Morand‐Beaulieu et al., 2017b; Murphy & Eddy, 2013). Language comprehension has also been suggested as a challenging area (Burd et al., 2008; Morand‐Beaulieu et al., 2017b), with a specific need identified for studies examining complex language comprehension (Murphy & Eddy, 2013). Difficulties on measures of high‐level and non‐literal language have been identified (De Nil et al., 2006; Morand‐Beaulieu et al., 2017b; Murphy & Eddy, 2013). Finally, past reviews have noted several factors that may relate to language and communication skills. These include motor skills, medication use, tic severity, social cognitive problems, inhibition and retrieval problems, the presence of autistic traits, co‐occurring ADHD and other co‐occurring conditions (Burd et al., 2008; De Nil et al., 2006; Morand‐Beaulieu et al., 2017b; Murphy & Eddy, 2013).

These prior reviews provide important insights into language and communication in TS; however, none reported a systematic search strategy and none provided the broad focus that characterises SLP models of language and communication. Moreover, while knowledge specific to childhood is relevant for developmentally‐focussed clinicians and researchers in speech‐language pathology, prior reviews have not focussed explicitly on children. The current scoping review differs from previous reviews in its focus on studies of children only, its intentional search for a wide range of measures of oral language and social communication, its use of a systematic search to capture relevant studies, and its focus on organising findings from a speech‐language pathology perspective. Some of the results of the current review overlap with findings from past reviews: Table 1 provides a summary of this overlap. To preview the results, the present search identified an additional 25 papers that were not described in previous reviews. Additionally, finding details that were not reported in previous reviews have been added in this scoping review.

**TABLE 1:** Previous reviews of language and communication in TS: Coverage and gaps.

	Number of studies cited (Language findings partially reported/not reported)	Number of studies cited (Complete language findings reported)	Number of studies *not* cited
Eddy (2021)	8	4	44
Morand‐Beaulieu et al. (2017b)	18	7	31
Murphy and Eddy (2013)	2	3	51
Burd et al. (2008)	1	0	55
De Nil et al. (2006)	9	1	46
*Total	23	8	25

* Excludes overlap across reviews.

Abbreviation: TS, Tourette syndrome.

### Purpose and objectives of the current scoping review

The purpose of the present review was to locate and describe the literature on oral language and social communication in children with TS. For our purpose, we define language as oral skills of expressive/receptive language, semantics, morphosyntax, narrative language and high‐level language. In keeping with Adams (2005) taxonomy of social communication, we define ‘social communication’ as skills of social interaction, social cognition, pragmatics (verbal and non‐verbal) and language processing. The scoping review does not address characteristics of tics, speech sound production or fluency in TS. For information on these topics, readers are directed to De Nil et al. (2006), Donaher (2006), Krah (2002) and Kwak and Jankovic (2002).

The objectives of this review are (1) to identify the scope and landscape of the current literature on oral language and social communication abilities in children with TS, (2) to present a summary of findings, and (3) to identify where gaps exist in this literature. A scoping review was chosen as the methodology as it can facilitate answering broad questions about the existing literature, such as ‘what is known about this concept?’ (Tricco et al., 2018, p. 467). A scoping review is also suited to mapping a literature that is scattered across several disciplines.

## METHODS

The protocol for this review was drafted using Preferred Reporting Items for Systematic Reviews and Meta‐analysis Extension for Scoping Reviews (PRISMA‐ScR, Tricco et al., 2018). We followed the framework introduced by Arksey and O'Malley in their seminal paper (2005) and the advances to this methodology introduced by Levac et al. (2010). We were informed by the systematic process for scoping reviews outlined in Peters et al. (2015).

### Literature search

Our search for literature began with a search strategy developed by the two authors and with the support of library services at the University of Alberta. A three‐step process as described by Aromataris and Riitano (2014) and Khalil et al. (2016) was employed, and several iterations were required before the full search could be carried out. First, a list of keywords relating to language and communication was developed based on terminology commonly used by SLPs and language researchers. Search terms were chosen to broadly capture literature about language and social abilities in people with TS. This list was refined through an initial review of literature in CINAHL and MEDLINE. Second, index terms were identified in each electronic database. Third, hand searching of reference lists of all papers that remained after applying our exclusion criteria was completed. See Appendix 1 for the full list of search terms and an example of our search strategy in MEDLINE.

To locate published documents, the following six databases were searched with adaptations to the search terms made as needed to meet database‐specific requirements: OVID MEDLINE (1946‐present), OVID EMBASE (1974‐present), EBSCOHost CINAHL Plus with fulltext (1937‐present), EBSCOHost ERIC, EBSCOHost Education Research Complete, EBSCOHost PsycINFO. In order to identify unpublished literature, two further databases were searched: Bielefeld Academic Search Engine (BASE) and ProQuest Dissertations & Theses. Searches were conducted on 9 February 2022. Results were exported into RefWorks. Duplicates were identified using the RefWorks duplicate tools and through hand searching. Duplicates were reviewed item by item and removed, leaving 865 records.

### Inclusion criteria

We included (1) scientific studies reporting primary data (i.e., information targeted at the general public was excluded; reviews and summaries were excluded, but their reference lists were searched for relevant papers), (2) studies that included children with TS (in some cases groups with co‐occurring conditions were present in the study; studies focussed on other tic disorders were not included), (3) Studies that focussed on children 6–18 years of age, and (4) studies that investigated concepts of oral linguistic ability or social communication.

Disagreements throughout the process were resolved by consensus. Only papers written in English were included. No specific date limits were applied and no exclusion criteria regarding methodology or number of participants were applied. Older research was included to be thorough and to allow for a full appraisal of what has been studied in the past.

#### Title/abstract screening

**FIGURE 1:**
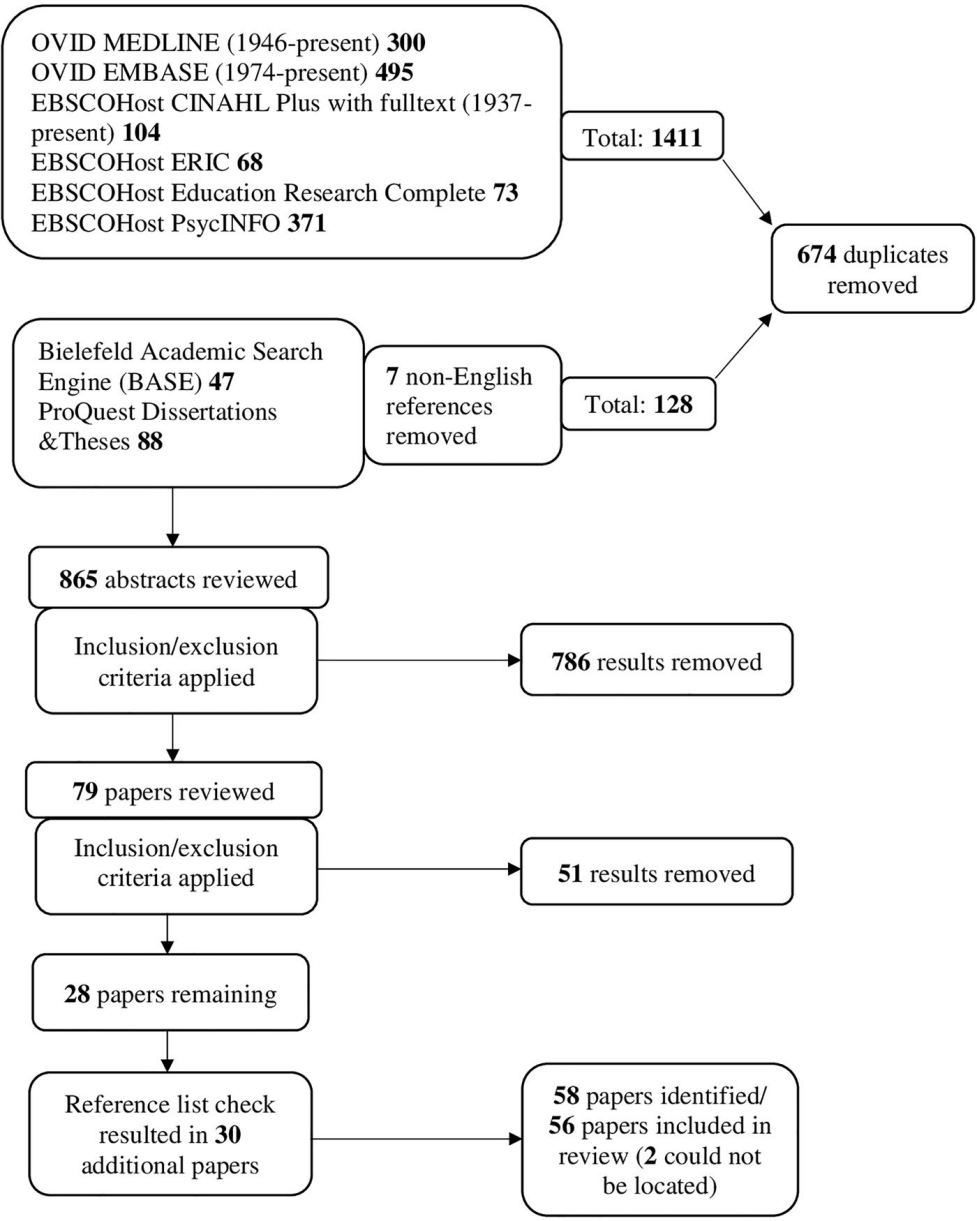
Flow of search for records.

The two authors independently reviewed all 865 titles and abstracts. Where there was ambiguity about any of the inclusion criteria, the paper was carried forward for a review of the full text. Inclusion and exclusion criteria were refined throughout the title and abstract review process. Search terms were broad enough to capture language studies involving the linguistic aspects of tics and studies involving reading and writing, however, these studies were excluded as we refined the boundaries of our topic area (oral language and social communication). At the title/abstract screening stage, 786 records were excluded, leaving 79 remaining. Figure 1 outlines the flow of search for records.

#### Full text review

For the remaining 79 articles, a full text review was completed independently by the two authors. Inclusion/exclusion criteria needed to be further defined at this stage. This was done by defining what constituted a study of children, what constituted a measure of oral language, and what constituted a measure of social communication.

Studies that involved both children and adults were included only if the population consisted of mostly children (>50% 19 years or younger) or if results for the paediatric population were presented separately.

Studies were included if they reported prevalence of language disorders, case descriptions of language development, receptive and/or expressive language ability, or performance on any task requiring language generation. Neuropsychological assessment tasks (i.e., measures of intelligence, memory and executive function) sometimes involved generating language. All subtests involving categorising, defining/describing and vocabulary knowledge/retrieval were included. Verbal IQ composite scores were also included in the review, as they are derived from language‐based tasks. Memory tasks that involved remembering a single word were not included; however, memory tasks requiring participants to reproduce a sentence or story were included, as they are similar to sentence repetition and story retell tasks used by S‐LPs. From executive functioning studies, we included verbal fluency tasks as they require language retrieval.

We used the Adams (2005) taxonomy to define the parameters of social communication measures. The Adams taxonomy includes (1) social interaction, (2) social cognition, (3) pragmatics (verbal and non‐verbal) and (4) language processing (i.e., receptive and expressive language). Therefore, all measures that fell within these four areas of social communication were included (note that language processing measures had already been included under the umbrella of ‘oral language’). All measures that provided an overall social communication score were included as well. Measures were included if they primarily investigated social interaction, defined by us as the child's ability to interact in social situations or motivation for social interaction. Under social interaction, we also included papers looking at ‘social behaviour’, ‘social incompetence’, ‘social motivation’, ‘socialisation’, and ‘social skills’ if the focus was commensurate with our definition of social interaction. Social skills and other social measures were not included if the focus was outside of this definition (e.g., involvement in social activities, quantity and quality of social relationships). We included measures that tested the child's ability to understand facial expressions and prosody but did not include simple facial recognition tasks. Studies measuring social cognitive abilities such as understanding mental states or theory of mind were also included. Finally, studies focussed on autism trait investigation were included, as these measures have a primary focus on social communication/interaction.

Twenty‐eight studies remained after this step. Through reviewing the reference lists of these 28 studies and 17 related articles, 30 additional papers were identified for inclusion, for a total of 58. Two of these papers could not be located, leaving 56 records in the review.

### Coding and summarising articles

Relevant information was extracted from the 56 studies using a spreadsheet developed and refined in collaboration by the authors. Data were fully charted by one reviewer with the second reviewer independently charting 20% of the data. Consistency was 96%. Most disagreements related to multi‐part studies where a control group was used for some studies and not others. One author coded them as ‘no control group’ and the other coded them as ‘control group’. All disagreements were resolved by consensus and remaining data were checked for similar differences. The data items that were charted included article characteristics (publication year, author, title and source), study design, population descriptors (total *N*, age, sex, diagnosis of study groups), oral language or social communication skill measured and results of oral language and social communication measures. There was considerable variability in the measurement tools used across studies and many of them come from disciplines outside of speech‐language pathology; therefore, specific tools were not charted. A focus on the skill measured (rather than the specific tool) facilitated the grouping of studies based on oral language and social communication constructs. In order to synthesise findings, a narrative summary was prepared by (1) grouping studies based on the skill measured; (2) further subdividing studies based on findings and the groups included; and (3) composing a summary of findings for each skill area by reporting on studies that did not consider co‐occurring conditions in their groupings, followed by reporting on studies that did consider co‐occurring conditions in their groupings.

## RESULTS

### Time frame and methods

Publication dates ranged from 1974 to 2019. Eleven studies were published before 1990, 16 between 1990 and 1999 and 29 after 2000. Studies were categorised as either descriptive (21 studies; 38%) or observational (35 studies; 63%). Descriptive studies included case studies of individuals with TS, studies comparing performance of children with TS relative to norms (normative studies), survey studies and mixed methods with qualitative interviews. Observational studies included comparisons of recruited samples of children with TS and control groups of children (correlational studies).

### Sources of records

The majority of the included records (47; 84%) were published in academic journals (date range = 1974–2019). Six (11%) of the studies were published in dissertations (date range = 1986–2006) and three (5%) were published in books (date range = 1982–1988). Thirty‐five different journals were listed for the 47 journal publications, leaving little overlap in the location of these articles. The most frequent journals were the *Journal of Child Psychology and Psychiatry and Allied Disciplines* (three articles; all published in 1988) and the *Journal of the American Academy of Child and Adolescent Psychiatry* (three articles; date range = 2000–2017). Thirty‐nine (83%) of the 47 articles were published in journals focussing on psychology/psychiatry, with an additional five (11%) focussing on paediatrics/development, one (2%) focussing on clinical linguistics, one (2%) focussing on autism and one (2%) focussing on genetics.

### Sample characteristics

The size of the TS sample recruited was >30 for 28 studies (50%) and <30 for 27 (48%). One study included multiple samples; one with more than 30 children and one with fewer than 30 children (2%). Participants across studies varied widely in age, from 6 to 67 years of age. Seven studies (12%) included individuals with TS over the age of 19 (in addition to the child participants; recall that our inclusion criteria required that >50% of participants be 19 years of age or younger). Sex breakdown of participants with TS most often included a greater number of males than females (in 53 studies or 95%; including seven studies with only male participants). One study (2%) included a greater number of females than males and two (4%) did not provide a sex breakdown.

### Synthesis of study findings

**FIGURE 2:**
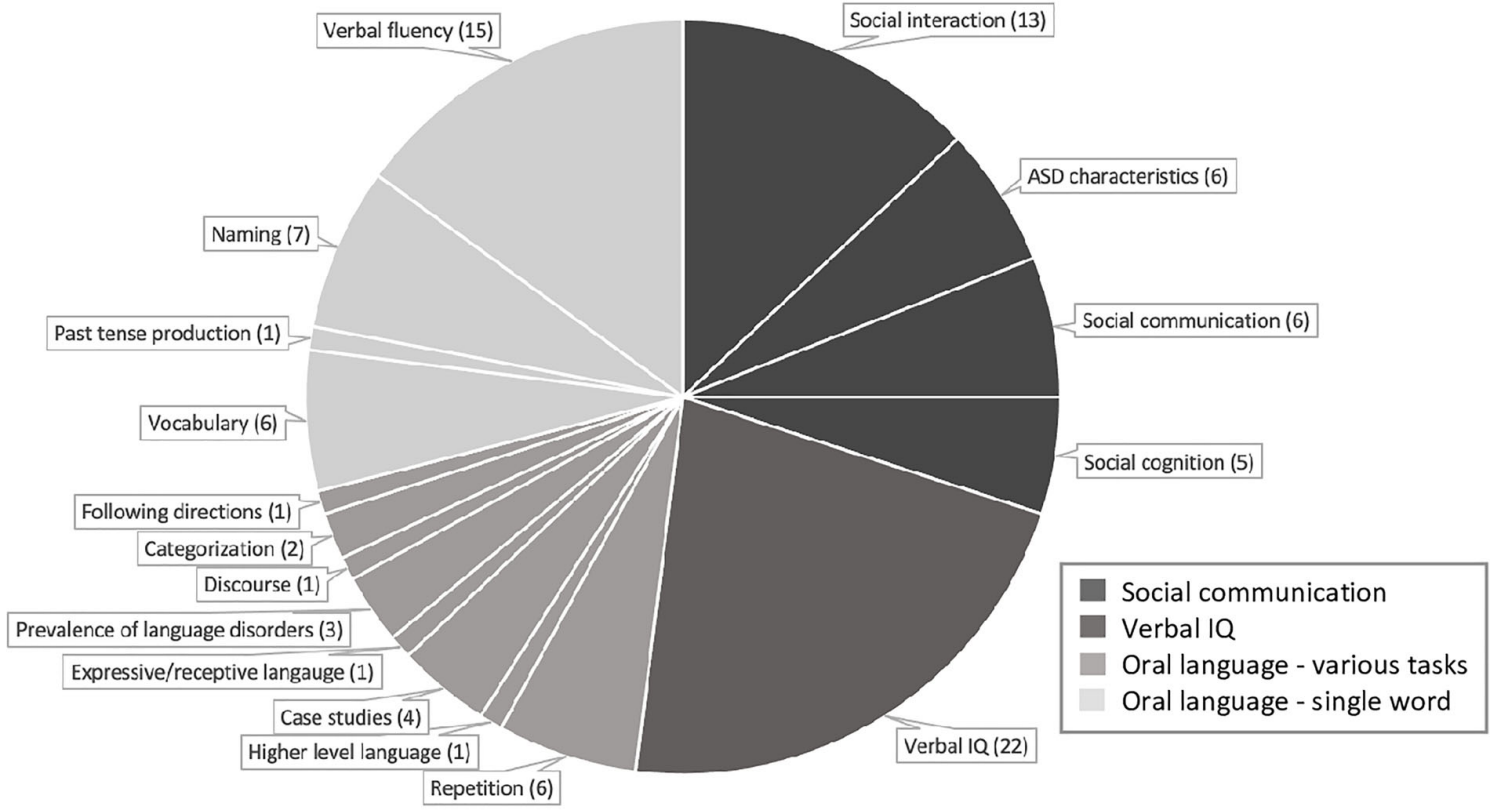
Oral language and social communication skills measured across studies. Abbreviation: ASD, autism spectrum disorder. [Colour figure can be viewed at wileyonlinelibrary.com]

Figure 2 presents a broad categorisation of oral language and social communication skills measured across studies. For a list of all studies included in the review and a detailed breakdown of the skill measured, see Appendix 2. In the narrative summary that follows, where studies described performance in relation to co‐occurring diagnoses, we include those findings. For a table of primary finding(s) organised by content area, see the Supplementary Materials. We have used a star (*) throughout our summary to indicate where individual findings have been previously presented in a published review of language in TS.

#### Oral language findings

Twenty‐three studies reported on the oral language abilities of children with TS. Of these, 16 studies had not previously been discussed in a review of language in TS. The prevalence of language disorders in children with TS was reported in three studies. Claussen et al. (2018) reported the prevalence of parent‐reported speech/language conditions as 30% in a US national sample of children with TS, compared to 4% in a sample from the general population. In the TS sample, 80% of children had a co‐occurring condition; 55% had co‐occurring ADHD. The occurrence of speech/language conditions was not reported in relation to these co‐occurring conditions. Cravedi et al. (2018) examined the prevalence of language delays/disorders in different clusters of children with TS. Prevalence was 45% in children with ‘complex’ TS (exhibiting multiple co‐occurring conditions), but only 3% in other clusters of children with TS. Spencer et al. (1998)* found that language disorders were present in 6% of children with TS only and 20% of children with TS and co‐occurring ADHD.

Receptive vocabulary was measured in five studies. Results all demonstrated average skills in children with TS (Bornstein et al., 1983; Ferrari et al., 1984; Jensen, 2004; Kalfayan Sweeten, 1997; Stokes et al., 1991). Jensen (2004) measured expressive vocabulary, reporting scores to be within the average range.

Pratt (2000) found no difference in confrontation naming accuracy between children with TS and a group of unaffected controls, whereas Hulbert (1986) found that the mean accuracy score for a group of children with TS on confrontation naming was more than one SD below the mean of the norming sample. Four studies examined naming speed. Walenski et al. (2007)* measured naming speed and accuracy for manipulated objects (e.g., hammer) and non‐manipulated objects (e.g., elephant). Children with TS and controls were equal in accuracy for both naming tasks and were equal in speed for non‐manipulated objects, but children with TS had faster naming speeds for manipulated objects. Jensen (2004) found that children with TS and children with TS and co‐occurring dyslexia had average speeds for a rapid automatic naming (RAN) task. Schuerholz et al. (1996)* found no difference in RAN speeds between groups of children with TS, TS and co‐occurring ADHD, and controls. Schuerholz et al. (1998) again found no difference between RAN speeds for children with TS compared to unaffected controls; but in contrast, children with TS and co‐occurring ADHD were significantly slower than children with TS only and controls. Children with ADHD only were also slower than the group with TS only.

Ludlow et al. (1982)* measured language skills using a collection of language expression tasks (verbal fluency, sentence construction, digit repetition, reading and writing names) and receptive comprehension tasks (specific tasks not reported). They found that children with TS scored significantly lower than controls on language expression tasks but not language comprehension tasks. How this pattern mapped to the specific tasks used was not further discussed. Hulbert (1986) found that children with TS scored, on average, more than one SD below the mean on a test of following directions. De Groot et al. (1997) found that children with TS plus co‐occurring conditions (OCD and ADHD) had significantly more trouble with a categorisation task than other groups with TS. Verté et al. (2005) found that children with TS had stronger scores on a categorisation task compared to children with autism. Walenski et al. (2007)* studied the accuracy and speed of past tense production. They found that there was no difference between children with TS and controls for accuracy, but children with TS completed the task faster than controls for regular past tense words and for some irregular forms.

Children's ability to recall stories, repeat sentences or repeat non‐words was measured in six studies. No difference between participants with TS and controls was found for the accuracy of story recall (Channon et al., 2003b*; Pratt, 2000; Takács et al., 2018), sentence repetition (Hulbert, 1986; Ludlow et al., 1982) or non‐word repetition (Dye et al., 2016*). Non‐word repetition speed was significantly higher in participants with TS compared to controls (Dye et al., 2016*).

Narrative language was evaluated using a fable task in a case series by Legg et al. (2005)*. This study performed a qualitative analysis of participant responses, concluding that ‘disrupted language processing and reduced language usage’ (p. 26) were present in 5/10 participants. Legg et al. (2005)* also evaluated specific areas of high‐level language by administering the Test of Language Competence (subtests include Understanding Ambiguous Sentences, Making Inferences, Recreating Sentences and Understanding Metaphoric Expression). Half of the participants obtained scores below the average range. Of the five adolescents who experienced challenges with these tasks, four had co‐occurring OCD, two had co‐occurring ADHD, and one had no co‐occurring conditions. Of those who did not experience challenges, all five had co‐occurring OCD, one had co‐occurring ADHD and one had co‐occurring borderline personality disorder.

Anecdotal descriptions of language development in children with TS were reported in four studies that presented a series of cases. Kalfayan Sweeten (1997) found that language skills were developmentally appropriate in an adolescent male with TS. Of five cases studied by O'Quinn and Thompson (1980)*, three were reported to have language formulation problems, two were reported to have word finding problems, and two were reported to have no language development problems. Thompson et al. (1979) reported on two children with TS, one with no language concerns, and one with language formulation, word finding and coherence difficulties. Comings and Comings (1991) reported delayed communication skill development in a group of children with TS who also had autism. Fourteen of the 16 participants showed difficulties. Comings and Comings also evaluated three children with TS who had a family member with autism. No challenges with language development were noted in those three children.

#### Verbal IQ findings

Twenty studies investigated the verbal intellectual abilities of children with TS using language‐based subtests or composite scores on tests of intellectual function. Nineteen of these studies had not been discussed in previous reviews. Eighteen studies found that children with TS, on average, scored within the normal range of verbal intellectual ability (Bornstein, 1990; Bornstein et al., 1991; Carter et al., 2000; Cravedi et al., 2018; Ferrari et al., 1984; Huckeba et al., 2008; Hagin & Kugler, 1998; Hulbert, 1986; Incagnoli & Kane, 1983; Jensen, 2004; Kalfayan Sweeten, 1997; Khalifa et al., 2010; O'Quinn & Thompson, 1980; Shapiro et al., 1974, 1988; Stokes et al., 1991; Thompson et al., 1979; Yeates & Bornstein, 1994). Lanser et al. (1993) found that children with TS scored significantly lower for verbal IQ compared to children with right hemisphere dysfunction.

Some studies have looked at verbal IQ in relation to co‐occurring diagnosis. Children with TS who had medium‐to‐low attention abilities (Huckeba et al., 2008) or co‐occurring ADHD (Carter et al., 2000), scored lower on measures of verbal IQ compared to a control group, but children with high attention abilities scored similarly to the control group. On the other hand, Yeates and Bornstein (1994) found that the verbal IQ of children with both TS and ADHD was not significantly different from children with TS only. De Groot et al. (1997) compared verbal IQ in groups of children with TS only and children with TS and co‐occurring conditions. Children with co‐occurring OCD or both OCD and ADHD had significantly lower scores, whereas children with TS and ADHD scored similarly to those with TS only.

#### Verbal fluency findings

Verbal fluency is a test of lexical access based either on the first letter of words or on the semantic category (semantics) of words. Although often used as a measure of neuropsychological function, verbal fluency is also strongly related to lexical ability (Whiteside et al., 2016). Fifteen studies presented results on the verbal fluency abilities of children with TS. The verbal fluency findings in five of these had not been previously reviewed.

Letter fluency scores were reported in 10 studies. Six of these reported no differences between the letter fluency scores of children with TS and control groups (Channon et al., 2003b*; Church et al., 2009*; Mahone et al., 2001*, 2002*; Schuerholz et al., 1996, 1998); three reported average normative scores (Harris et al., 1995*; Hulbert, 1986; Jensen, 2004). Brookshire et al. (1994) found that children with TS scored lower than their unaffected siblings. The effect of having co‐occurring conditions on letter fluency scores was considered in four studies. Letter fluency scores of children with TS who also had ADHD did not differ from children with TS only (Channon et al., 2003b; Harris et al., 1995; Mahone et al., 2002), controls (Channon et al., 2003b; Mahone et al., 2002; Schuerholz et al., 1996*, 1998*), children with TS who also had OCD (Channon et al., 2003b) or children with ADHD only (Harris et al., 1995; Mahone et al., 2002). Schuerholz et al. (1996)* and Schuerholz et al. (1998)* found that children who only had TS scored lower on letter fluency than children who had TS and ADHD. Mahone et al. (2001) compared children with ADHD only to children with TS only, finding no difference.

For category fluency, five studies reported scores for children with TS. No difference between the category fluency scores of children with TS and controls were reported by Mahone et al. (2001)*, Mahone et al. (2002), Schuerholz et al. (1996)* and Schuerholz et al. (1998). Harris (1995)* found that, on average, children with TS scored within the normal range for their age but children with TS and ADHD scored lower than expected for their age. Brand et al. (2002)* found that children who also had ADHD performed more poorly than children with TS only. Harris et al. (1995)* found no significant difference between performance for children with TS only, ADHD only and TS with co‐occurring ADHD. Mahone et al. (2001) found that children with ADHD only performed more poorly than children with TS.

Four studies of verbal fluency did not differentiate between category and letter fluency. The scores of children with TS did not differ from controls (Drury et al., 2018; Verté et al., 2005), children with TS and ADHD (Drury et al., 2018), children with TS and autism (Verté et al., 2005; described by authors as high functioning autism) or autism only (Verté et al., 2005). On the other hand, one study found that children with TS had higher verbal fluency scores than children with TS and OCD (regardless of whether or not they had ADHD; De Groot et al., 1997*). Drury et al. (2018) found that children with TS and ADHD had lower verbal fluency scores than controls. Khalifa et al. (2010)* found that children with TS scored lower than would be expected based on age norms (scores were below the 25th percentile).

#### Findings related to social communication

Findings related to social communication are presented here in four groupings, chosen according to the Adams (2005) taxonomy and adjusted based on the types of studies located. The groupings include, overall social communication, social interaction, social cognition and Autism traits. No studies were grouped under pragmatics (Adams’ third category) and language processing studies are presented above (Adams’ fourth category).

Six studies reported on the overall social communication abilities of children with TS using caregiver questionnaires. None of these findings had been previously presented in reviews of language in TS (although some related skills had been reported on—i.e., social interaction). Eapen et al. (2019), Güler et al. (2015) and Verté et al. (2005) found statistically significant increases in social communication problems for groups with TS compared to controls. The samples in these studies experienced co‐occurring conditions representative of the TS population. Eapen et al. (2019) found that social communication was more of a challenge in a group with autism than in a group with TS. Darrow et al. (2017) found that children with TS plus OCD had stronger social communication scores compared to children with TS only and children with TS plus ADHD. Darrow et al. (2017) and Sukhodolsky et al. (2003) found no significant difference between children with TS and unaffected controls, but found that children with TS and co‐occurring ADHD had significantly more challenged social communication scores compared to unaffected controls. Sukhodolsky et al. (2003) also studied children with ADHD only and found that they had more challenges compared to children with only TS but similar scores to children with TS plus ADHD. Darrow et al. (2017) found that the children with TS only and TS plus ADHD had more challenges than the children with TS plus OCD and ADHD. Dykens et al. (1990) found that social communication was not a particular weakness for children with TS relative to other intellectual, academic and adaptive skills measured. Conversely, Darrow et al. (2017) found that two groups with TS (TS with OCD and TS with both ADHD and OCD) had higher scores on the social communication measure compared to controls.

Social interaction was measured in 13 studies. These studies measured either motivation to interact socially or skill in social interaction using caregiver questionnaires. Findings in 11 of these studies had never been reported in a previous review. Dykens et al. (1990) reported social interaction as a weakness for children with TS in relation to other adaptive skills. Eighty‐five percent of Kadesjö and Gillberg's (2000) sample were reported to have significant problems interacting with same‐age peers. Eapen et al. (2019) reported social interaction problems compared to a control group. Darrow et al. (2017)*, Gorman et al. (2010) and Güler et al. (2015)* found that children's scores were significantly poorer on measures of social interaction compared to control groups of unaffected children. On the other hand, Bawden et al. (1998) found that when children with TS were compared to a control group with a chronic condition (diabetes mellitus) there was no significant difference. Social interaction problems correlated significantly with problematic home behaviour, lowered quality of life and poorer family relations (Marek, 2006), as well as higher overall TS severity (Hulbert, 1986).

Three of these studies considered social interaction in children with TS and ADHD separately from children who had TS only. Carter et al. (2000) and Sukhodolsky et al. (2003) found that children with TS and co‐occurring ADHD scored significantly lower on social interaction measures compared to children with only TS and compared to unaffected controls. When comparing children with TS only and unaffected controls, Carter et al. (2000) and Sukhodolsky et al. (2003) did not find significant differences in social interaction skills. Sukhodolsky et al. (2003) found that children with only TS had better social interaction scores than children with ADHD only, but the TS plus ADHD group scored similarly to the ADHD only group. Hoekstra et al. (2004) found that the presence of co‐occurring ADHD predicted social insight problems but not social contact problems. Gallina (1989) compared social interaction in children with TS, children with ADHD and controls. There was a significant difference among the groups with the ADHD group scoring lowest, the TS group scoring second lowest, and the control group scoring highest. A post hoc analysis was not completed in order to determine which pairwise differences were significant.

Five studies examined skills of social cognition in children with TS. Only one new study (previously unreviewed) was added. Guler et al. (2015)* found that children with TS had poorer social cognition scores compared to controls on a parent questionnaire. Drury et al. (2018)* also investigated comprehension of sarcasm that was presented in simulated scenarios. The authors tested both direct sarcasm (e.g., remarking ‘[you're] the best cook in the world’ in response to burned toast) and indirect sarcasm (e.g., remarking ‘I'll hire you in my restaurant’ in response to burned toast; examples from Drury et al. (2018), p. 491). When sarcasm was presented directly there was no difference across TS and control groups. When sarcasm was presented indirectly performance was significantly poorer in children with TS compared to unaffected controls. Drury et al. (2018) also investigated children's ability to answer questions about mental states of people in these sarcastic scenarios. The TS and control groups performed similarly on these questions; however, mental state identification was lower for children with TS who performed more poorly on the indirect sarcasm measure. Rajendran et al. (2005)* compared comprehension of sarcasm in children with TS to children with autism. No significant difference was found. Rajendran et al. (2005) also compared comprehension of figures of speech and recognition of inappropriate requests in children with TS to children with autism. Children with autism had significantly lower scores. A case series published by Baron‐Cohen and Robertson (1995) used two false belief tasks and a deception task to evaluate whether or not six children (one with TS, two with autism and three with TS and autism) had a grasp of theory of mind. It was concluded that the child with TS had intact theory of mind, while those with autism (or TS and autism) did not. Drury et al. (2012)* found that children with TS could discriminate and name emotions in facial expressions and vocal prosody with similar accuracy to unaffected controls. Compared to a control group, children with TS and ADHD had trouble recognising angry prosody and judging whether or not it was congruent with the message being said.

Six studies reported on autism traits in children with TS. Four of these had not been previously reviewed. Percentages of children in TS samples exhibiting autism traits were reported by Darrow et al. (23%; 2017)*, Kadesjö and Gillberg (20%; 2000) and Wadman et al. (26%; 2016). The presence of autism traits was found to be significantly lower in groups of children with TS compared to children with autism (Eapen et al., 2019; Verté et al., 2005). When compared to a typically developing control group; however, children with TS scored significantly higher on measures of autism traits (Eapen et al., 2019; Güler et al., 2015*).

## DISCUSSION

This scoping review was undertaken with the purpose of locating existing studies about the oral language and social communication abilities of children with TS and describing the composition of that literature. The objectives were to identify the scope of the current literature, summarise the findings presented in studies, and identify gaps in this literature. In keeping with the purposes of a scoping review, we focussed on drawing conclusions about the state of the literature, rather than evaluating findings. Nonetheless, in some cases, study limitations were evident with regard to publication date, sample size, methodology and the tasks used to measure oral language and social communication skills.

### Current state of the literature

We found 56 records that measured skills of oral language or social communication in children with TS. The majority of these papers and books came from the fields of psychology/psychiatry. Only a single article, a case series of adolescents with TS, was published in a speech‐language pathology‐focussed journal (Legg et al., 2005, *Clinical Linguistics*).

#### Oral language

There was some variety in the skills measured across oral language literature, however, the majority of studies examined verbal IQ or verbal fluency, as we might expect when language is investigated from a psychology or psychiatry standpoint. We did not find any studies investigating language ability at the level of conversation (i.e., using conversational language sample analysis). Also, no single study within this review provided a detailed description of the language system involving multiple domains (i.e., form, use and content) and modalities (i.e., expressive and receptive), using, for example, the omnibus tests common to SLP clinical practice. Rather, isolated aspects of language have frequently been reported within the context of questions about neuropsychological and social aspects of TS.

#### Social communication

With regard to the scope of social communication skills measured across studies, social interaction has been measured most widely. Although there are several studies measuring social interaction, they have not observed the interaction skills of children with TS directly, looked at direct effects of tics on social interaction encounters, surveyed the points of view of the children themselves about their interactions or involved other familiar interactants such as peers. Measures of social communication were almost entirely composed of parent questionnaires and reported only a mean total score, giving little clue as to where reported functional communication challenges lie. Social cognition has received some research attention thus far as well, but several social cognitive skills remain unstudied. Rates of children with TS who exhibit traits of autism are well established in this literature.

### Oral language and social communication in children with TS: Summary of findings

We summarise the findings of the literature with caution given the small number of studies that have examined language skills beyond verbal IQ tests and verbal fluency, and the fact that many of the reviewed studies have elements that limit interpretation or generalisation (e.g., case descriptions, studies lacking detailed reporting). On the whole, the reviewed literature points to verbal IQ and verbal fluency in TS as being consistent with typical expectations. Single‐word vocabulary appears to be an area of strength. The literature suggests elevated rates of language disorder among children with complex TS (i.e., with co‐occurring diagnoses). There is preliminary evidence of high‐level language challenges in some children with TS (Legg et al., 2005). There are also some very preliminary hints from case studies and more dated research that challenges exist in following directions, language formulation and word finding (Hulbert, 1986; O'Quinn & Thompson, 1980; Thompson et al., 1979). When considering social communication, there is evidence for elevated presence of autism traits in children with TS, though lower than what is generally seen in autism. There is also evidence for difficulties in social communication, social interaction and social cognition, including understanding indirect sarcasm and recognising message congruence for an angry tone of voice (Drury et al., 2012, 2018).

#### Impact of co‐occurring diagnoses

Whenever studies reported on the relationship between co‐occurring diagnoses and outcomes, we have described those findings. Performance on various tasks was sometimes poorer for groups with co‐occurring conditions. This pattern was seen in studies that investigated overall prevalence of language disorders, social interaction, recognising angry tone of voice, categorisation, naming speed and verbal IQ; however, it is important to note that not all studies showed this pattern and not all studies provided data on co‐occurring conditions. Careful documentation of the presence of co‐occurring conditions and their relationship to outcomes will be an important consideration in future language and communication research with children who have TS. Developing our understanding of oral language and social communication concerns that present in TS only (i.e., ‘pure’ TS) is of interest given past findings in social communication, social cognition and high‐level language and given that we know problems can exist in areas of social, academic and cognitive development. On the other hand, since approximately 80% of individuals with TS have co‐occurring conditions (Martino & Leckman, 2013), future research should be careful about focusing exclusively on pure TS, as findings will represent only a small subset of the population. Further, findings may not represent the type of child who would most likely be referred to the SLP (i.e., the ‘complex’ child).

### Refining and extending conclusions about oral language and social communication

Consistent with previous reviews covering aspects of oral language and social communication in TS, this review has found that children with TS exhibit autism‐like characteristics, speeded language processing and developmentally appropriate verbal fluency skills. High‐level language challenges have also been identified; however, we emphasise that although these findings have repeatedly been presented in past reviews, they remain preliminary in nature.

When considering the literature on oral language in children with TS from a speech‐language pathology perspective, we must be cautious about some conclusions that have been previously presented regarding expressive language, receptive language and language development more generally in TS. The terms ‘expressive’ and ‘receptive’ have been used inconsistently in past reviews on the subject and have sometimes been used to refer to other language‐related skills. Some studies citing weak expressive language in children with TS have measured verbal fluency, speech sound perception or reading and writing (e.g., Brookshire, 1994; Bornstein et al., 1990; Ludlow, 1982; cited in de Nil, 2006, Eddy, 2021; Murphy & Eddy, 2013). Although verbal fluency represents an aspect of expressive language, the SLPs view of expressive language is much broader, usually involving a comprehensive look at elements of semantic, morphological, syntactic and pragmatic development, at the levels of the single word, sentence and larger discourse units. Despite this, verbal fluency findings have been relied upon heavily in past conclusions about expressive language and overall language in TS (e.g., Channon et al., 2003b; Mahone et al., 2001; cited by de Nil et al., 2006). Additionally, one review suggested that language comprehension concerns may exist secondary to social cognitive challenges in TS (Morand‐Beaulieu et al., 2017b). While social cognitive challenges are very likely to have a complex effect on functional language use, we currently cannot rule out challenges in basic receptive language skills in children with TS. With five studies demonstrating average receptive vocabulary, and two older studies showing challenges in following directions and performance on an unknown set of receptive language tasks (Bornstein et al., 1983; Ferrari et al., 1984; Hulbert, 1986; Jensen, 2004; Kalfayan Sweeten, 1997; Ludlow 1982; Stokes et al., 1991), we have barely scratched the surface of receptive language evaluation in this population.

Discussion of social communication in TS is mounting in the literature: several authors of past reviews have identified it as an area of concern in TS and literature on social cognitive challenges in adults has become well established (e.g., Eddy et al., 2010a, 2010b, 2011, 2016, 2017; Eddy & Cavana, 2015). Some researchers are querying if social communication could be a central component of the disorder since individuals with TS often exhibit socially inappropriate behaviour and have challenges on tasks involving social reasoning and social interpretation (See Albin, 2018; Eddy, 2021; Eddy & Cavanna, 2013; Eddy et al., 2011). Only a few studies of social cognition in children exist currently and, as previously stated (e.g., Eddy et al., 2011; Morand‐Beaulieu et al, 2017b), findings indicate that these challenges are subtle. Differences in social interpretation seem to appear in some contexts only (i.e., indirect sarcasm and understanding angry tone). The current review is the first to present the full range and depth of studies on social communication in children, and through employing the Adams (2005) taxonomy of social communication, has begun to organise this literature from a speech‐language pathology point of view. This mapping indicates that social communication problems exist across at least two of the four domains of social communication (Adams, 2005): social interaction and social cognition.

Although average or near average intellectual abilities are frequently discussed in TS literature, verbal IQ findings in children had not been reported previously with regard to language development. We identified this skill as a strength for children with TS after reviewing all of the available studies on the topic. Similarly, past reviews have not looked specifically at vocabulary, categorisation or ability to recall/repeat language, all areas that we have identified as strengths.

### Considerations for future research

Future reviews evaluating literature on oral language and social communication in TS should pay particular attention to study power, task difficulty and tool validity. A future review on this topic may choose to employ a systematic review methodology in order to formally evaluate the strength of evidence. To further broaden our knowledge‐base, it would be beneficial for future studies to consider studies of children with other tic disorders, such as chronic motor vocal tic disorder and provisional tic disorder. Furthermore, the language abilities of adults with TS are also of interest and should be considered in future reviews.

From this mapping of the existing literature on oral language, we suggest that there is still a need for comprehensive study of language abilities in children with TS with a focus on coverage across domains and modalities and attention to high‐level language abilities and discourse contexts. Larger sample sizes with control are needed to further preliminary findings in the literature. Additionally, there is a need for information about communication abilities in natural contexts where all of the factors that present for children with TS are at play (e.g., social perceptions, memory, tics). There is also a need for information about a larger range of social cognitive skills in children, while keeping in mind the importance of differing task difficulty. Finally, future studies should focus on more detailed descriptions of social communication skills and social experiences, moving beyond measurement of autism‐specific traits.

### Potential role of the SLP in support of children with TS

Language development in children with TS has not been investigated in much detail in the speech‐language pathology literature thus far. The summary provided here indicates that SLPs will be more likely to see children with complex forms of TS (e.g., those with TS and ADHD) on their caseloads. SLPs should pay attention to screening children with TS for language disorders, investigating social communication and social interaction development and investigating narrative language, language formulation and high‐level language skills. Treatment strategies may involve directly targeting areas of challenge and/or providing appropriate accommodations (e.g., extra time to complete activities, modified activities, extra teacher instruction). As they get older, young people with TS may benefit from education and coaching to advance self‐advocacy skills. Given the vast literature on neuropsychological development in children with TS, interdisciplinary collaboration could further clarify the role of the SLP in providing interventions for children experiencing challenges in their language and social communication development.

### Limitations

When reporting conclusions about the current research findings, an important limitation to consider is that the quality of the evidence has not been systematically evaluated. Although the inclusion of research from prior to 2000 enabled a full review of existing literature, the age of some studies constrains the reader's ability to interpret findings as reporting standards and the definition and diagnostic process for TS have changed over time. Finally, this review focussed on skills ‘within the child’, but it is important to recognise that contextual factors such as environment and social and self‐stigma associated with tics may play an important role in the communication experiences of children with TS (e.g., Malli et al., 2016).

## CONCLUSION

The present review revealed 56 studies investigating oral language and/or social communication abilities in children with TS. Although a large number of studies exist on this topic, some areas still remain under investigated. When considering the social and academic challenges that children with TS exhibit and the social cognitive challenges that adults with TS exhibit, we suggest that more comprehensive research into the language and communication skills of children with TS is needed. Collaboration with other disciplines, such as psychology, neuropsychology and psychiatry would be of benefit in future research and clinical endeavours as these disciplines have a well‐established literature of research with this clinical population.

## CONFLICT OF INTEREST STATEMENT

The authors declare no conflicts of interest.

## Supporting information

Supporting Information

## Data Availability

The data that support the findings of this study are available from the corresponding author upon reasonable request

## Appendix 1

Search Strategy and Dates Searched

Database: OVID MEDLINE (1946‐present)

Date: November 7, 2019

Searched by: Angela Feehan

Number of results: 270

Search strategy:

1. (Language skills or language characteristics or high level language impairment or discourse impairment or normal communicative performance or language profiles or ‘concreteness of language’ or poor language formulation or frontal cortex language involvement or language development or language disorders or grammar or language or linguistic knowledge or idiosyncratic knowledge or mental lexicon or regular past tense or irregular past tense or linguistics or language tests or learning disabilities or developmental dyslexia or dyslexia or learning disorders or social decision making or social communication or social communication deficits or social communication questionnaire or sarcastic remarks or mentalising or pragmatic language or sarcasm or ‘theory of mind’ or social cognition).mp. [mp=title, abstract, original title, name of substance word, subject heading word, floating sub‐heading word, keyword heading word, organism supplementary concept word, protocol supplementary concept word, rare disease supplementary concept word, unique identifier, synonyms]
2. limit 1 to English language
3. Child Language/ or Language Development/ or Language/ or Communication Disorders/ or Language Disorders/ or Language Development Disorders/ or Speech Language Pathology/ or Semantics/ or Vocabulary/ or Metaphor/ or Narration/ or Social Communication Disorder/ or ‘Theory of Mind’/ or Social Skills/ or Nonverbal Communication/ or Facial Expression/ or Social Perception/ or Interpersonal Relations/ or Literacy/ or Writing/ or Reading/ or Agraphia/ or Dyslexia/ or Learning Disorders/ or Language Tests/ or ‘Rehabilitation of Speech and Language’/ or Language Therapy/
4. limit 3 to English language
5. 2 or 4
6. Tourette Syndrome/ or tourette*.mp.
7. limit 6 to English language
8. 5 and 7

The following databased were searched on February 9th, 2022:

OVID EMBASE (1974‐present)

EBSCOHost CINAHL Plus with full text (1937‐present)

EBSCOHost ERIC

EBSCOHost Education Research Complete

EBSCOHost PsycINFO

Bielefeld Academic Search Engine (BASE):

ProQuest Dissertations & Theses

## Appendix 2 Scoping Review Studies

**Table:**

	Lead author	Year	Study type	Participant groups	Skills measured
1	Baron‐Cohen	1995	Descriptive	TS	Theory of mind
				ASD	
				TS+ASD	
2	Bawden	1998	Observational	TS	Social interaction
				Diabetes mellitus	
3	Bornstein	1990	Descriptive	TS	Verbal intelligence
4	Bornstein	1983	Descriptive	TS	Receptive vocabulary
					Verbal intelligence
5	Bornstein	1991	Descriptive	TS	Verbal intelligence
6	Brand	2002	Observational	TS	Verbal fluency
				TS+ADHD	
7	Brookshire	1994		Observational	TS
					Normal siblings
					Arithmetic disability
8	Carter	2000	Observational	TS	Verbal intelligence
				TS+ADHD	Social interaction
				Control	
9	Channon	2003	Observational	TS	Verbal fluency
				TS+ADHD	Story recall
				TS+OCD	
				Control	
10	Church	2009	Observational	TS	Verbal fluency
				Control	
11	Claussen	2018	Descriptive	TS	Prevalence of language problems
12	Comings	1991	Observational	TS with an autistic relative	Language development in cases
				ASD+TS	
13	Cravedi	2018	Descriptive	Pure TS	Verbal intelligence
				Complex TS	Expressive and receptive language
				TS+Attention & writing problems	
14	Darrow	2017	Observational	TS	Social communication
				TS+ADHD	Social interaction
				TS+OCD	Prevalence of autistic traits
				TS+ADHD+OCD	
				Family members of people with TS	
15	De Groot	1997	Observational	TS	Verbal intelligence
				TS+ADHD	Verbal fluency
				TS+OCD	Categorisation
				TS+ADHD+OCD	
16	Drury	2018	Observational	TS	Sarcasm comprehension
				TS+ADHD	Verbal fluency
				Control	(Non)mentalistic questions
17	Drury	2012	Observational	TS	Emotion perception
				TS+ADHD	
				Control	
18	Dye	2016	Observational	TS	Non‐word repetition
				Control	
19	Dykens	1990	Observational	TS	Social communication
				TS+ADHD	
20	Eapen	2019	Observational	TS	Social communication
				ASD	Social interaction
				Control	Autistic traits
21	Ferrari	1984	Descriptive	TS	Receptive vocabulary
					Verbal intelligence
22	Gallina	1989	Observational	TS	Social interaction
				ADHD	
				Control	
23	Gorman	2010	Observational	TS	Social interaction
				Control	
24	Güler	2015	Observational	TS	Social communication
				Control	Social interaction
					Social cognition
					Autistic traits
25	Hagin	1988	Descriptive	TS	Verbal intelligence
26	Harris	1995	Observational	TS	Verbal fluency
				ADHD	
				TS+ADHD	
27	Hoekstra	2004	Descriptive	TS	Social interaction
28	Huckeba	2008	Observational	TS	Verbal intelligence
				Control	
29	Hulbert	1986	Descriptive	TS	Social interaction
					Naming
					Sentence repetition
					Following directions
					Verbal intelligence
					Verbal fluency
30	Incagnoli	1983	Descriptive	TS	Verbal intelligence
31	Jensen	2004	Observational	TS	Verbal fluency
				Dyslexia	Receptive vocabulary
				Tics+Dyslexia	Expressive vocabulary
32	Kadesjö	2000	Descriptive	TS	Social interaction
					Autistic traits
33	Kalafayan Sweeten	1997	Descriptive	TS	Receptive vocabulary
					verbal intelligence
					Language development in a case
34	Khalifa	2010	Observational	TS	Verbal intelligence
				Control	Verbal fluency
35	Lanser	1993	Observational	TS	Verbal intelligence
				Right hemisphere dysfunction	
36	Legg	2005	Descriptive	TS	Higher‐level language
				Control	Narrative language
37	Ludlow	1982	Observational	TS	Language expression
				Control	Language comprehension
					Sentence repetition
38	Mahone	2002	Observational	TS	Verbal fluency
				ADHD	
				TS+ADHD	
				Control	
39	Mahone	2001	Observational	TS	Verbal fluency
				ADHD	
				Control	
40	Marek	2006	Descriptive	TS	Social interaction
41	O'Quinn	1980	Descriptive	TS	Language development in cases
					Verbal intelligence
42	Pratt	2000	Observational	TS	Story recall
				Control	Naming
43	Rajendran	2005	Observational	TS	Social cognition (sarcasm, figures of speech, inappropriate requests)
				ASD (Asperger syndrome)	
				Control	
44	Schuerholz	1996	Observational	TS	Naming
				TS+ADHD	Verbal fluency
				TS+QueryADHD	
				Unaffected siblings	
45	Schuerholz	1998	Observational	TS	Naming
				TS+ADHD	Verbal fluency
				ADHD	
				Control	
46	Shapiro	1988	Observational	TS	Verbal intelligence
				TS+ADHD	
				Psychiatric referral	
				Control	
47	Shapiro	1974	Descriptive	TS	Verbal intelligence
48	Spencer	1998	Observational	TS	Prevalence of language disorder
				TS+ADHD	
				ADHD	
				Psychiatric referral	
				Control	
49	Stokes	1991	Descriptive	TS	Receptive vocabulary
					Verbal intelligence
50	Sukhodolsky	2003	Observational	TS	Social communication
				ADHD	Social interaction
				TS+ADHD	
				Control	
51	Takács	2018	Observational	TS	Story recall
				Control	
52	Thompson	1979	Descriptive	TS	Verbal intelligence
					Language development in cases
53	Verté	2005	Observational	TS	Verbal fluency
				ASD (high functioning)	Categorisation
				TS+ASD (high functioning)	Social communication
				Control	Autistic traits
54	Wadman	2016	Descriptive	TS	Autistic traits
55	Walenski	2007	Observational	TS	Past tense production
				Control	Naming
56	Yeates	1994	Observational	TS	Verbal intelligence
				TS+ADHD	

Abbreviations: ADHD, attention‐deficit/hyperactivity disorder; ASD, autism spectrum disorder; OCD, obsessive compulsive disorder; TS, Tourette syndrome.
