# Supplementary material for: A scoping review of oral language and social communication abilities in children with Tourette syndrome
Source: Int J Lang Commun Disord. 2023 Sep 4;59(1):143–64. doi: 10.1111/1460-6984.12949 (PMC13507563; doi:10.1111/1460-6984.12949)
Supplement: Supplementary file 1 [file JLCD-59-143-s001.docx]

**Supplementary Table 1**

*Key Study Findings*

| **Sub-domain** | **Findings that suggest challenges relative to control group** | **Findings comparing pure TS to complex TS** | **Other findings** |
| --- | --- | --- | --- |
| **Social communication** |  |  |  |
| Social interaction | **TS < Control**  (Darrow et al., 2017; Gorman et al., 2010; Gallina, 1989; Güler et al., 2015)  **TS+ADHD < Control**  (Carter et al., 2000a; Sukhodolsky et al., 2003)  **A weakness in relation to other adaptive skills.** (Dykens et al., 1990)  **85% have problems interacting with peers.**  (Kadesjö & Gillberg, 2000)  **Social interaction problems elevated**  (Eapen et al., 2019)  **Co-occurs with home behaviour problems, lower quality of life, poorer family relations**  (Marek, 2006)  **Co-occurs with high tic severity** (Darrow, Hirschtritt, et al., 2017) **and high TS severity** (Hulbert, 1986) | **TS+ADHD < TS**  (Carter et al., 2000; Sukhodolsky et al., 2003) | **TS = Control**  (Carter et al., 2000a; Sukhodolsky et al., 2003)  **TS = Diabetes Mellitus**  (Bawden et al., 1998)  **ADHD < TS**  (Gallina, 1989; Sukhodolsky et al., 2003)  **ADHD = TS+ADHD**  (Sukhodolsky et al., 2003) |
| Social communication | **TS < Control**  (Güler et al., 2015; Verté et al., 2005)  **TS+ADHD < Control**  (Darrow et al., 2017; Sukhodolsky et al., 2003)  **HFA+TS < Control**  (Verté et al., 2005)  **Children with TS had lower social communication scores than controls (significance not tested). Scores for children with ASD were lower still.**  (Eapen et al., 2019) | **TS < TS+OCD+ADHD**  (Darrow et al., 2017)  **TS < TS+OCD**  (Darrow et al., 2017)  **HFA+TS < TS**  (Verté et al., 2005)  **TS+ADHD < TS+OCD+ADHD**  (Darrow et al., 2017)  **TS+ADHD < TS+OCD**  (Darrow et al., 2017)  **TS = TS+ADHD**  (Darrow et al., 2017; Sukhodolsky et al., 2003)  **TS+OCD+ADHD = TS+OCD**  (Darrow et al., 2017) | **TS = Control**  (Darrow et al., 2017; Sukhodolsky et al., 2003)  **TS = ADHD**  (Sukhodolsky et al., 2003)  **TS+ADHD = ADHD**  (Sukhodolsky et al., 2003)  **HFA < TS**  (Verté et al., 2005)  **HFA = HFA+TS**  (Verté et al., 2005)  **Control < TS+OCD**  (Darrow et al., 2017)  **Control < TS+OCD+ADHD**  (Darrow et al., 2017)  **Not a weakness for children with TS or TS+ADHD relative to other intellectual, academic, and adaptive skills.**  (Dykens et al., 1990) |
| Autistic traits (reversed so that lower scores indicate greater presence of traits) | **TS < Control**  (Eapen et al., 2019; Güler et al., 2015)  **Present in 23% of children with TS.**  (Darrow et al., 2017).  **Present in 20% of children with TS.**  (Kadesjö & Gillberg, 2000)  **Present in 26% of children with TS.**  (Wadman et al. 2016b). | **HFA+TS < TS**  (Verté et al., 2005) | **ASD < TS**  (Eapen et al., 2019)  **HFA < TS**  (Verté et al., 2005)    **HFA+TS = HFA**  (Verté et al., 2005) |
| Social cognition | General  **TS < Control**  (Güler et al., 2015)  Indirect sarcasm  **TS < Control**  (Drury et al., 2018)  Anger recognition:  **TS+ADHD < Control**  (Drury et al., 2012)  **Three cases with TS+ASD had challenges with theory of mind.**  (Baron-Cohen & Robertson, 1995)  **Comorbid ADHD predicts social insight problems** (Hoekstra et al., 2004) |  | Direct sarcasm:  **TS = Control**  (Drury et al., 2018)  **TS = AS**  (Rajendran et. al., 2005)  General emotional (and anger) recognition:  **TS = Control**  (Drury et al., 2012)  Figures of speech:  **AS < TS**  (Rajendran et. al., 2005)  Recognition of inappropriate requests:  **AS < TS**  (Rajendran et. al., 2005)  **One case of a child with TS had no problems with theory of mind.**  (Baron-Cohen & Robertson, 1995) |
| **Oral language** |  |  |  |
| Prevalence of oral language delays | **30% in a sample of children with TS (compared to 4% in control sample).**  (Claussen et al., 2018)  **45% in children with “complex” TS (exhibiting multiple comorbidities)**  (Cravedi et al., 2018)  **20% in children with TS+ADHD**  (Spencer et al, 1998) | **3% in children with TS (without complex comorbidities).**  (Cravedi et al., 2018)  **6% in children with TS (without ADHD)**  (Spencer et al, 1998) |  |
| Receptive language | Following directions  **Children with TS scored below average.**  (Hulbert, 1986) |  | Language comprehension (tasks unknown)  **TS = Control**  (Ludlow et al., 1982)  Receptive vocabulary  **average in children with TS.**  (Bornstein et al., 1983; Ferrari et al., 1984; Jensen, 2004; Kalfayan Sweeten, 1997; Stokes et al., 1991) |
| Expressive language | Language expression (oral and written tasks)  **TS < Control**  (Ludlow et al., 1982) |  | Accuracy of past tense production:  **TS = Control**  (Walenski et al., 2007)  Speed of past tense production:  **Control < TS**  (Walenski et al., 2007)  **Expressive vocabulary average in children with TS.**  (Jensen, 2004) |
| Naming accuracy | **Below average in children with TS.**  (Hulbert, 1986) |  | **TS = Control**  (Dye et al., 2016; Pratt, 2000) |
| Naming speed | General:  **TS+ADHD < Control**  (Schuerholz et al., 1998) | General:  **TS+ADHD < TS**  (Schuerholz et al., 1998)  **TS = TS+ADHD**  (Schuerholz et al., 1996) | General:  **TS = Control**  (Schuerholz et al., 1996; Schuerholz et al., 1998)  **TS+ADHD = Control**  (Schuerholz et al., 1996)  **ADHD < TS**  (Schuerholz et al., 1998)  Manipulated objects:  **Control < TS**  (Walenski et al., 2007)  Non-manipulated objects:  **TS = Control**  (Walenski et al., 2007)  **Average for children with TS and TS+Dyslexia.**  (Jensen, 2004) |
| Categorization |  | **TS+ADHD < TS**  (De Groot et al., 1997)  **TS+OCD < TS**  (De Groot et al., 1997)  **TS+ADHD+OCD < TS**  (De Groot et al., 1997) | **ASD < TS**  (Verté et al., 2005) |
| Story recall |  |  | **TS = Control**  (Channon et al., 2003; Pratt, 2000; Takács et al., 2018) |
| Sentence repetition |  |  | **TS = Control**  (Hulbert, 1986; Ludlow et al., 1982) |
| Non-word repetition |  |  | Accuracy:  **TS = Control**  (Dye et al., 2016)  Speed:  **Control < TS**  (Dye et al., 2016). |
| Narrative language | **Qualitative analysis concluding “disrupted language processing and reduced language usage” (p. 26) in 5/10 subjects.**  (Legg et al., 2005) |  |  |
| Higher level language | **Half of cases with TS were below average for making inferences, recreating sentences, and understanding metaphoric expression.**  (Legg et al., 2005) |  |  |
| Case study reports of language development | **3/5 cases had language formulation problems and 2/5 cases had word finding problems.**  (O’Quinn & Thompson, 1980)  **One of two cases had language formulation, word finding, and coherence problems.**  (Thompson et al., 1979)  **14/16 cases with TS+ASD had language delays.**  (Comings & Comings, 1991) |  | **2/5 cases had no language concerns.**  (O’Quinn & Thompson, 1980)  **One of two cases had no language concerns.**  (Thompson et al., 1979)  **2/16 cases with TS+ASD had no language concerns.**  (Comings & Comings, 1991)  **A single case with developmentally appropriate language.**  (Kalfayan Sweeten, 1997) |
| **Verbal intelligence** |  |  |  |
| Verbal intelligence | **TS < RHD**  (Lanser et al., 1993)  **TS+ADHD < Control**  (Carter et al., 2000; Yeates & Bornstein, 1994) | **TS+OCD < TS**  (De Groot et al., 1997)  **TS+OCD+ADHD < TS**  (De Groot et al., 1997)  **TS = TS+ADHD**  (Carter et al., 2000b; De Groot et al., 1997)  **TS+ADHD = TS+OCD+ADHD = TS+OCD**  (De Groot et al., 1997) | **TS scores within normal**  (Bornstein, 1990; Bornstein et al., 1991; Carter et al., 2000a; Cravedi et al., 2018; Ferrari et al., 1984; Huckeba et al., 2008; Hagin & Kugler, 1998; Hulbert, 1986; Incagnoli & Kane, 1983; Jensen, 2004; Kalfayan Sweeten, 1997; Khalifa et al., 2010; O’Quinn & Thompson, 1980; Shapiro et al., 1974; Shapiro et al., 1988; Stokes et al., 1991; Thompson et al., 1979; Yeates & Bornstein, 1994)  **TS = Control**  (Carter et al., 2000b; Huckeba et al., 2008) |
| **Verbal fluency** |  |  |  |
| Letter fluency | **TS < Unaffected siblings**  (Brookshire et al., 1994) | **TS < TS+ADHD**  (Schuerholz et al., 1996; Schuerholz et al., 1998)  **TS = TS+ADHD**  (Channon, Crawford, et al., 2003; Harris et al., 1995; Mahone et al., 2002)  **TS = TS+OCD**  (Channon, Crawford, et al., 2003)  **TS+ADHD = TS+OCD**  (Channon, Crawford, et al., 2003) | **TS = Control**  (Channon et al., 2003; Church et al., 2009; Mahone et al., 2002; Mahone et al., 2001; Schuerholz et al., 1996; Schuerholz et al., 1998)  **TS+ADHD = Control**  (Channon, Crawford, et al., 2003; Mahone et al., 2002; L. J. Schuerholz et al., 1996; Linda J. Schuerholz et al., 1998)  **TS = Arithmetic disability**  (Brookshire et al., 1994)  **TS+OCD = Control**  (Mahone et al., 2002)  **ADHD < TS+ADHD**  (Schuerholz et al 1998)  **ADHD = TS**  (Mahone et al., 2001)  **TS scores within normal**  (Hulbert, 1986; Jensen, 2004) |
| Categorical fluency | **TS+ADHD scores lower than expected based on norms**  (Harris et al., 1995) | **TS+ADHD = TS**  (Harris et al., 1995)  **TS = TS+ADHD**  (Schuerholz et al., 1996; Schuerholz et al., 1998; Mahone et al., 2002) | **TS = Control**  (Mahone et al., 2001; Mahone et al., 2002; Schuerholz et al., 1996; Schuerholz et al., 1998)  **TS+ADHD = Control**  (Mahone et al., 2002; Schuerholz et al., 1996; Schuerholz et al., 1998)  **ADHD < TS**  (Mahone et al., 2001)  **TS scores within normal** (Harris et al., 1995) |
| Verbal fluency unspecified | **TS+ADHD < Control**  (Drury et al., 2018)  **TS scores lower than expected based on norms** (Khalifa et al., 2010) | **TS+OCD** < **TS**  (De Groot et al., 1997)  **TS+OCD+ADHD** < **TS**  (De Groot et al., 1997)  **TS = TS+HFA**  (Verté et al., 2005)  **TS+ADHD = TS+OCD = TS+OCD+ADHD**  (De Groot et al., 1997) | **TS = Control**  (Drury et al., 2018; Verté et al., 2005)  **TS = TS+ADHD**  (De Groot et al., 1997; Drury et al., 2018)  **TS+HFA = HFA**  (Verté et al., 2005)  **TS = HFA**  (Verté et al., 2005)  **TS+HFA = Control**  (Verté et al., 2005) |

HFA - High functioning autism

OCD - Obsessive compulsive disorder

ADHD - Attention deficit hyperactivity disorder

ASD – Autism Spectrum Disorder

AS – Asperger’s Syndrome

RHD – Right Hemisphere Dysfunction
